# Supplementary material for: Physical activity among older adults with multimorbidity: Evidence from a population-based health survey
Source: PLoS One. 2024 Jan 2;19(1):e0296460. doi: 10.1371/journal.pone.0296460 (PMC10760873; doi:10.1371/journal.pone.0296460)
Supplement: Attachment — Submitted filename: Dear Editor.docx [file pone.0296460.s002.docx]

Dear Editor,

Thanks’ for the invitation and the opportunity to review the manuscript titled "physical activity among older adults with multimorbidity: Evidence from a population-based health survey," submitted to the journal PLOS One.

The study aims to estimate the prevalence of multimorbidity and physical activity, as well as to analyze the relationship between meeting recommended levels of physical activity and multimorbidity, according to the characteristics of older adults residents in São Paulo, Brazil.

Overall, the text shows a clear structure and an appropriate methodological design for the research question. The findings point to interesting results regarding the prevalence of multimorbidity and sufficient levels of physical activity. However, in the inferential analyses of association, there appears to be some difficulty in interpreting and establishing the relevance of the findings for the field of public health.

In this regard, in order to consider the manuscript for publication, I understand that the following are need: a) an improved justification of the study's importance; revising the presentation of the association results between physical activity and the number of morbidities, as there is an association with two morbidities but not with three, four, or five morbidities. However, an older adult with three, four, or five morbidities is considered to have multimorbidity. Therefore, this presentation needs to be more detailed; c) finally, the discussion does not establish plausible biological relationships to support the findings. For example, elderly individuals aged 80 or older with multimorbidity have a prevalence of being active four times higher than those without morbidities. What is the practical implication of this result? Was this result expected? Are there other international literature data that support these findings?

In the evaluation, I provide other elements that contribute to my decision.

Cassiano R Rech

Reviewer
