# Supplementary material for: Physical activity among older adults with multimorbidity: Evidence from a population-based health survey
Source: PLoS One. 2024 Jan 2;19(1):e0296460. doi: 10.1371/journal.pone.0296460 (PMC10760873; doi:10.1371/journal.pone.0296460)
Supplement: Attachment — Submitted filename: Dear Authors.docx [file pone.0296460.s003.docx]

Dear Authors,

Congratulations on the writing of the article "physical activity among older adults with multimorbidity: Evidence from a population-based health survey," submitted to the journal PLOS One.

The study aims to estimate the prevalence of multimorbidity and physical activity, as well as to analyze the relationship between meeting recommended levels of physical activity and multimorbidity, according to the characteristics of older adults residents in São Paulo, Brazil.

Major Concerns:

The study's introduction shows important information, but it does not clearly state the research problem. The authors argue that there is a relationship between physical activity and morbidities, implying that more older adults more active (those who meet recommendations) have a lower chance of having multimorbidity. Or is the hypothesis that older adults with morbidities are more active? The authors need to clarify this hypothesis. Additionally, they should present results from studies that have already tested this relationship to demonstrate the knowledge gap they are addressing. The fact that the study is conducted in a high-middle-income country (Brazil) cannot be the sole justification for the research.

In the methods section, the authors have chosen to perform an association analysis with the variable multimorbidity (two or more morbidities), but this information is presented categorically according to the number of morbidities. These results show that only having two morbidities was associated with the prevalence of being active, suggesting that among elderly individuals with morbidities, the prevalence of being active is higher than in those without or with one morbidity. Would this result be expected? The authors do not explain why individuals with four or more morbidities, for example, do not have a lower prevalence of being active. Wouldn't it be expected that individuals with more morbidities would have a lower prevalence of being active? This is not clear in the article.

Minor Concerns:

Page 2, line 38 – There is no need to present the classification of insufficient level of physical activity.

Page 2, lines 43 and 44 – Adjust the text to "two chronic diseases" and "three or more chronic diseases” no use 2 or 3.

Page 3, line 77 – The authors mention that one of the factors contributing to the increase in the prevalence of insufficient levels of physical activity is the transition from a professional life to retirement. However, there are many other factors, and this is just one of them. If other factors are not described, I suggest excluding this argument, as it is not mentioned further in the text.

Page 3, line 78 – The information about the number of deaths attributed to physical inactivity is out of place. This information could be placed where problems related to physical inactivity are discussed.

Page 4, line 85 – The authors mention that in the study by Zho et al., both regular and irregular physical activity were associated with chronic diseases. The text is confusing; this is in comparison to those who are inactive. The authors can clarify this point.

Page 4, line 90 – The study's focus is on physical activity, so it is suggested to avoid including sedentary behavior. This is another outcome that is not the focus of the study and providing this information might confuse the readers.

Method:

Page 5, line 114 – Mention that the data were collected before the COVID-19 pandemic.

Results:

Page 8, Table 1 – Display the unit of measurement for per capita income.

Page 8, line 200 – The authors could test the relationship between physical activity and multimorbidity, as this was the justification outlined in the introduction.

Page 8, Table 2 – Standardize the number of decimal places in the table (e.g., 63.7 (54.6-72.0)).

Page 9, Table 3 – The authors should consider that the group aged 80 has only six inactive elderly individuals without multimorbidity, which is a very low proportion. How might this impact the results? Could the existing association be due to the fact that almost all elderly individuals over 80 have multimorbidity?

Discussion:

Page 10, line 217 – The authors state that physical activity was maintained for individuals with three or more chronic conditions. This result is not presented.

Page 10, line 230 – One of the main study results shows that elderly individuals with two chronic conditions have a 37% higher prevalence of sufficient levels of physical activity. There is no justification for the presented results. Methodological differences are important, but beyond them, what explanations could account for this relationship?

Page 12, line 298 – The authors need to explore how the results can be applied in the field of public health. How can the results be considered by public policy planners? It is also suggested to remove the reduction of sedentary behavior, as this behavior was not evaluated in the study.
